# Newly Synthesized Melphalan Analogs Induce DNA Damage and Mitotic Catastrophe in Hematological Malignant Cancer Cells

**DOI:** 10.3390/ijms232214258

**Published:** 2022-11-17

**Authors:** Anastazja Poczta, Piotr Krzeczyński, Maksim Ionov, Aneta Rogalska, Udo S. Gaipl, Agnieszka Marczak, Dorota Lubgan

**Affiliations:** 1 Department of Medical Biophysics, Faculty of Biology and Environmental Protection, University of Lodz, 141/143 Pomorska St., 90-236 Lodz, Poland; 2 Department of Pharmacy, Cosmetic Chemistry and Biotechnology, Team of Chemistry, Łukasiewicz Research Network—Industrial Chemistry Institute, 8 Rydygiera St., 01-793 Warsaw, Poland; 3 Department of General Biophysics, Faculty of Biology and Environmental Protection, University of Lodz, 141/143 Pomorska St., 90-236 Lodz, Poland; 4 Translational Radiobiology, Department of Radiation Oncology, University Hospital Erlangen Friedrich-Alexander-Universität Erlangen-Nürnberg, Universitätsstr. 27, 91054 Erlangen, Germany

**Keywords:** blood cancer, DNA damage γH2AX, drug structure modification, melphalan, mitotic catastrophe

## Abstract

Myeloablative therapy with highdoses of the cytostatic drug melphalan (MEL) in preparation for hematopoietic cell transplantation is the standard of care for multiple myeloma (MM) patients. Melphalan is a bifunctional alkylating agent that covalently binds to nucleophilic sites in the DNA and effective in the treatment, but unfortunately has limited therapeutic benefit. Therefore, new approaches are urgently needed for patients who are resistant to existing standard treatment with MEL. Regulating the pharmacological activity of drug molecules by modifying their structure is one method for improving their effectiveness. The purpose of this work was to analyze the physicochemical and biological properties of newly synthesized melphalan derivatives (EE-MEL, EM-MEL, EM-MOR-MEL, EM-I-MEL, EM-T-MEL) obtained through the esterification of the carboxyl group and the replacement of the the amino group with an amidine group. Compounds were selected based on our previous studies for their improved anticancer properties in comparison with the original drug. For this, we first evaluated the physicochemical properties using the circular dichroism technique, then analyzed the zeta potential and the hydrodynamic diameters of the particles. Then, the in vitro biological properties of the analogs were tested on multiple myeloma (RPMI8226), acute monocytic leukemia (THP1), and promyelocytic leukemia (HL60) cells as model systems for hematological malignant cells. DNA damage was assessed by immunostaining γH2AX, cell cycle distribution changes by propidium iodide (PI) staining, and cell death by the activation of caspase 2. We proved that the newly synthesized derivatives, in particular EM-MOR-MEL and EM-T-MEL, affected the B-DNA conformation, thus increasing the DNA damage. As a result of the DNA changes, the cell cycle was arrested in the S and G2/M phases. The cell death occurred by activating a mitotic catastrophe. Our investigations suggest that the analogs EM-MOR-MEL and EM-T-MEL have better anti-cancer activity in multiple myeloma cells than the currently used melphalan.

## 1. Introduction

Hematopoietic malignancies are a heterogeneous group of cancers that affect the bone marrow, blood, and lymph nodes. They account for 10% of all annual cancer deaths worldwide. The second most common hematologic malignancy, after non-Hodgkin’s lymphoma, is multiple myeloma (MM). The disease is most often manifested by hypercalcemia, renal failure, anemia, and bone changes [1,2,3].

The therapeutic arsenal for MM patients includes drugs with various mechanisms of action, such as alkylating agents (melphalan, bendamustine), immunomodulatory drugs (thalidomide, lenalidomide, pomalidomide), histone deacetylase inhibitors (vorinostat, panobinostat), proteasome inhibitors (bortezomib, carfilzomib, ixazomib), and monoclonal antibodies (daratumumab, isatuximab) [4]. Treatment strategies vary depending on the patient’s age, comorbidities, disease stage, cytogenetic parameters, and other factors. For the treatment of primary patients under the age of 70 years without serious comorbidities, the treatment program includes high-dose melphalan (MEL), followed by autologous stem cell transplantation [5,6].

The cytotoxic properties of melphalan are due to its alkylating properties [7]. The alkylation of DNA takes place in two steps, involving the first and then the second chloroethyl group in the melphalan molecule, which create a highly reactive aziridinium intermediate cation [4,8]. Then, the resulting reactive intermediates rapidly alkylate guanine or adenine sites of the DNA to form the primary monoadduct. After the formation of the primary DNA monoadduct, the second chloroethyl group acts in this same manner. However, the secondary cyclization reaction is much slower than the first. The 2′-hydroxyethyl derivatives are the predominant DNA changes resulting from melphalan treatment, because DNA–DNA cross-links play a key role in the biological activity of melphalan. As a result of alkylation, many different DNA adducts can be formed: monoadduct (if the reactive intermediate interacts with the H_2_O molecule, the adduct remains a DNA monoadduct), DNA–protein cross-linking (if it reacts with an adjacent protein), intra-strand cross-linking (if the reaction takes place with guanine or adenine of the same DNA strand as the initial monoadduct), and inter-strand cross-linking (if the interaction is with the opposite strand of DNA) [9,10,11]. DNA–DNA cross-linking is usually associated with cell death or loss of a chromosome. The intra- or inter-strand lesions may be promutagenic or fatal [10].

Although using a high concentration of MEL in myeloablative therapy in preparation for hematopoietic cell transplantation remains the standard of care for MM patients [4], relapses are still common, with periods of remission becoming shorter and shorter. Therefore, new approaches are urgently needed for patients who are resistant to existing therapies [12].

Regulating the pharmacological activity of drug molecules by modifying their structure is one method of improving their effectiveness. The chemical structure of a drug determines its physicochemical properties, absorption, distribution, metabolism, excretion, and toxicity, which ultimately affects its pharmacological activity [13]. In our previous studies [14,15], we have shown that esterification of the carboxyl group is necessary to improve the effectiveness of MEL. In addition, replacing the amino group with an amidine group containing a thiomorpholine, indoline, or morpholine residue in the structure increases the properties of the drug. The new analogs synthesized by us have been designed to have good drug-like properties, which was confirmed by in silico studies [15]. In vitro studies of the biological properties, including the cytotoxic, pro-apoptotic, and genotoxic properties, showed the new derivatives to have improved properties. Simultaneously, a decreased cytotoxic effect in the tested derivatives was observed against peripheral blood mononuclear cells (PBMC) [14,15]. Such derivatives could potentially be of therapeutic importance. However, they require additional tests, including elucidating the mechanism of action that leads to their cytotoxic and genotoxic effects. The purpose of this work was to analyze the physicochemical and biological properties within our scientific framework of five synthesized derivatives (EE-MEL, EM-MEL, EM-MOR-MEL, EM-I-MEL, EM-T-MEL) in comparison with the original drug, melphalan. In particular, we focused on analyzing their effects on DNA and on understanding the cell death pathway in three cancer cell lines: multiple myeloma (RPMI8226), acute monocytic leukemia (THP1), and acute promyelocytic leukemia (HL60).

## 2. Results

### 2.1. Chemical Modifications of the Melphalan Molecule Alter the Conformation of B-DNA

**Figure 1:**
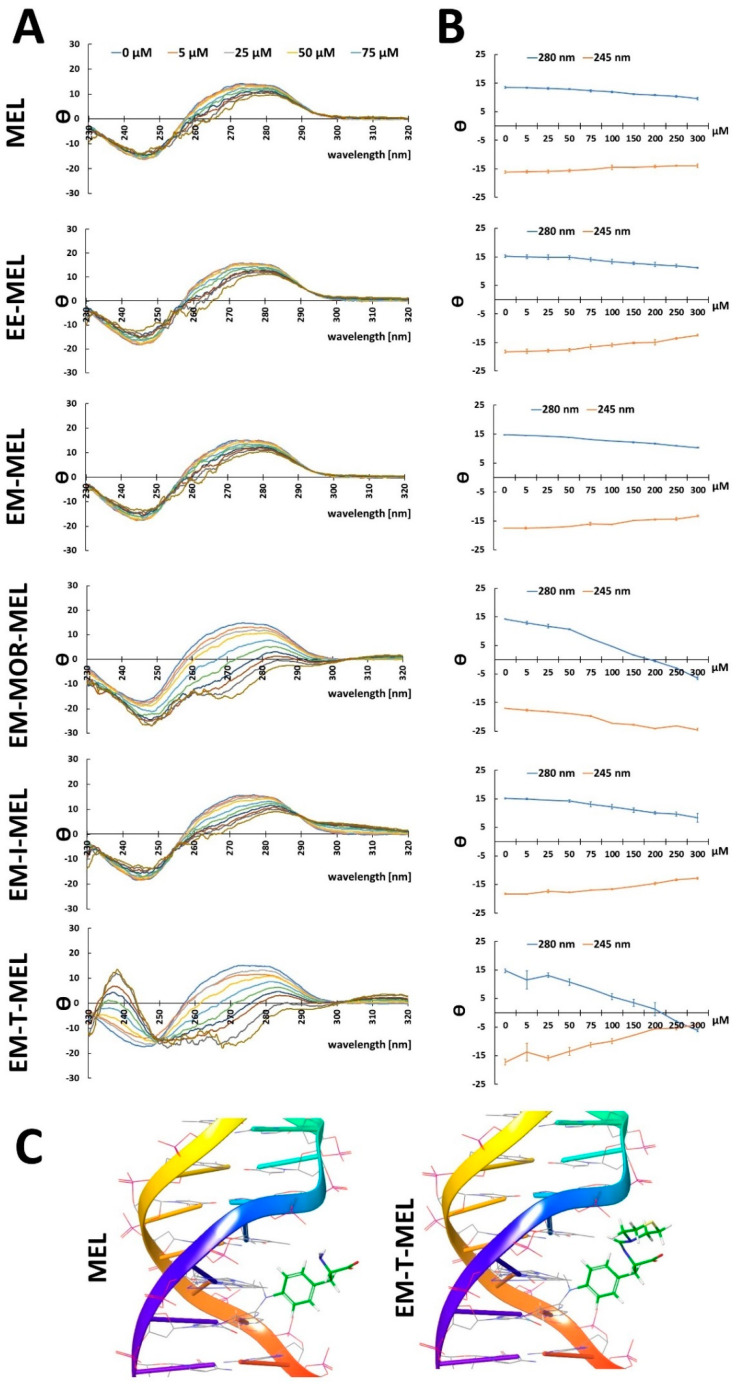
Chemical modifications of the melphalan molecule affect the B-DNA structure. (**A**) The CD spectra of DNA in the presence of MEL, EE-MEL, EM-MEL, EM-MOR-MEL, EM-I-MEL, and EM-T-MEL. (**B**) Changes in θ/θ0 parameter at λ = 245 nm and λ = 280 nm in the presence of MEL, EE-MEL, EM-MEL, EM-MOR-MEL, EM-I-MEL, and EM-T-MEL. Results are the mean ± standard deviation (SD), *n* = 3. (**C**) Visualizations showing the binding site to DNA of the original drug MEL and the most potent derivative EM-T-MEL. The visualizations were created in PyMOL (Schrödinger, Inc.). Computer simulation was performed for one DNA molecule and one ligand molecule.

To analyze the ability of melphalan and the investigated melphalan derivatives (EE-MEL, EM-MEL, EM-MOR-MEL, EM-I-MEL, EM-T-MEL) to affect DNA secondary structures, the circular dichroism (CD) technique was applied. This method is widely used to study the conformation of biomolecules, and is based on the difference in absorption of left and right circularly polarized light. The obtained spectrum was typical for the B-formation of DNA (as described in [16,17]) with characteristic peaks: positive around λ = 280 nm and negative around λ = 245 nm. The positive band appears as a result of a stacking interaction between the DNA nitrogen bases, while the negative band indicates the right-handedness of the B-DNA double helix [18]. CD spectra were recorded in the λ = 230–320 nm range. The CD results are presented as molar ellipticity (θ) in deg × cm^2^ × dmol^−1^ (Figure 1A). All the drugs had an impact on the DNA. The greatest changes in ellipticity were observed for EM-T-MEL and EM-MOR-MEL.

The conformational transition of DNA can be traced by changes in the positions and intensities of the CD spectral peaks (Figure 1A,B) [19]. The shift in the peak from 280 nm to higher wavelengths (EM-T-MEL: 287 nm; EM-MOR-MEL: 288 nm), followed by a decrease in intensity, indicated that the DNA form changed from form A to form B [20]. The ellipticity of the DNA changed with increasing derivative concentration (5–300 µM). At the highest concentration of EM-T-MEL, the CD peak intensity at λ = 280 nm decreased and reached a minimum at λ = 268 nm. An increase in intensity was determined at λ = 245 nm, reaching a maximum at λ = 238. The EM-MOR-MEL derivative caused relevant changes in ellipticity as well. This compound caused a decrease in the intensity of the CD peaks and two negative CD peaks (for the highest concentration: λ = 271 nm and λ = 248 nm). The ability of MEL and its derivative EM-T-MEL to form complexes with DNA was confirmed and also studied by computer modeling (Figure 1C).

### 2.2. Melphalan and Its Derivatives Exhibit a Negative Zeta Potential

**Figure 2:**
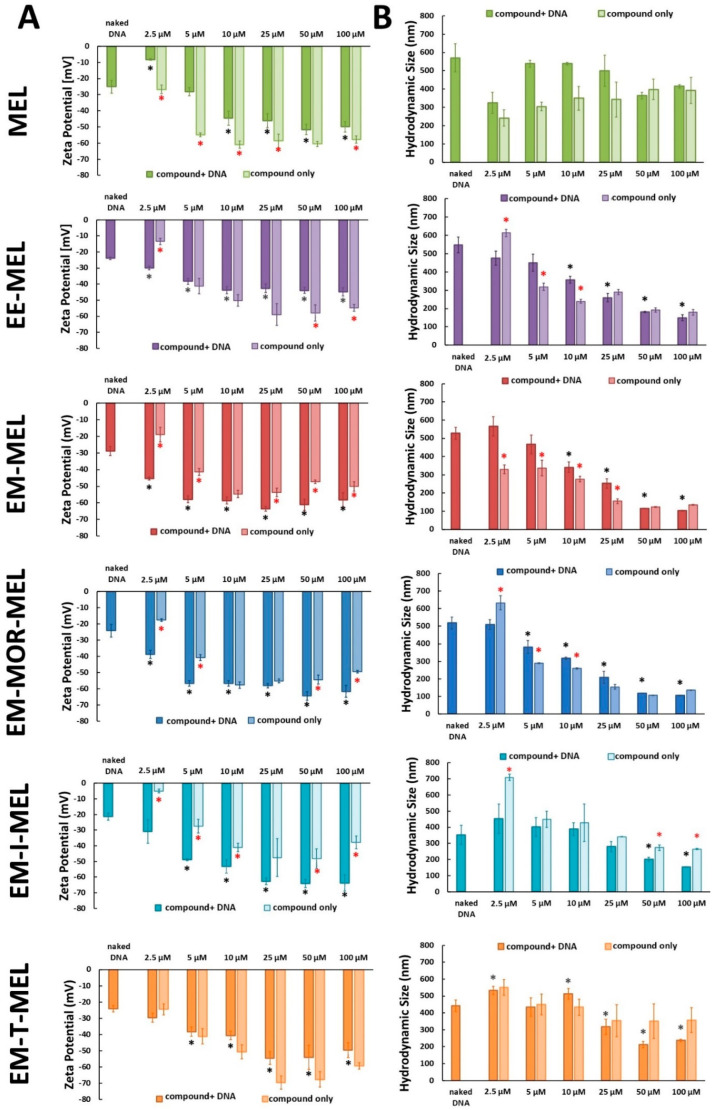
(**A**) Zeta potential of the original drug and all tested analogs with and without DNA. (**B**) The hydrodynamic size of MEL, EE-MEL, EM-MEL, EM-MOR-MEL, EM-I-MEL, and EM-T-MEL with and without DNA. Results are the mean ± SD. (black asterisks *) Statistically significant differences between a sample containing only DNA vs. a sample containing DNA and the tested compound, *p* < 0.05; (red asterisks *) statistically significant differences between a sample containing DNA and the tested compound vs. a sample containing only tested compound, *p* < 0.05, n = 3.

Zeta potential measurements provide information about the surface charge of tested compounds and their complexes with DNA (Figure 2A). The zeta potential of naked DNA was negative (approximately −23 mV). MEL and all tested analogs were negatively charged as well. The zeta potential of the original MEL molecule ranged from −27 mV (2.5 µM) to −58 mV (10–100 µM). The addition of negatively charged DNA increased the zeta potential to values ranging from −8 mV (2.5 µM) to −44 mV (10 µM). The EM-T-MEL derivative at a concentration of 25 µM had the lowest zeta potential (approximately −70 mV). The addition of DNA resulted in a significant increase in the zeta potential, by up to −53 mV. For the other compounds, we also observed low zeta potential values for the samples with and without DNA. Adding increasing concentrations of the compounds to the DNA caused a gradual but significant decrease in the zeta potential. EM-MEL and EM-MOR-MEL in the already lowest concentration of 5 µM, MEL at 10 µM, and the remaining derivative at 25 µM induced increased (due to higher concentrations) modifications in the zeta potential.

The hydrodynamic diameters of the tested compounds and their complexes with DNA were measured by the dynamic light scattering (DLS) technique (Figure 2B). We did not observe an increase in the average size of the complex when the tested compounds were added to DNA compared to the sample containing DNA only. The DLS results showed that melphalan and its tested analogs did not cause an increase in the DNA hydrodynamic diameter.

### 2.3. Tested Derivatives Induce DNA Damage Detected by γH2AX Analysis

**Figure 3:**
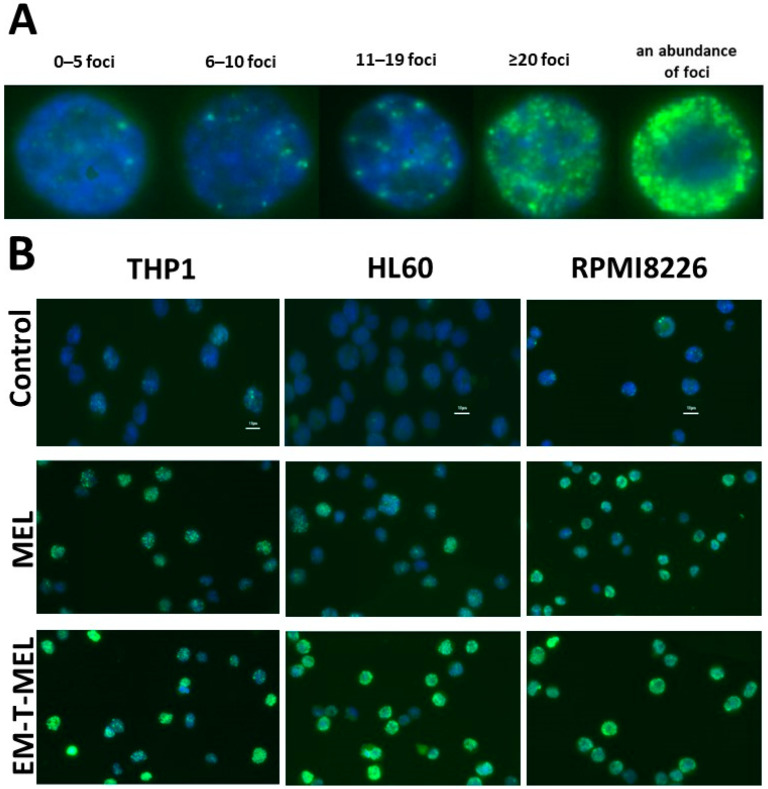
(**A**) Representative photos showing RPMI8226 cells with a number of foci (0–5; 6–10; 11–19; ≥20; an abundance of foci) per cell. (**B**) Detection and localization of γH2AX histone foci in THP1, HL60, and RPMI8226 cells based on the highly specific antigen–antibody binding reaction. Sample images after 24 h of incubation. Cells were incubated with a phospho-(Ser139) mouse monoclonal IgG specific anti-H2AX antibody, and next with Alexa Fluor TM 488 goat anti-mouse IgG secondary antibody as described in the Materials and Methods section. The cell nuclei were stained with 4′,6-diamidino-2-phenylindole (DAPI) solution. The cells were visualized under a fluorescence microscope. Magnification: 400x, scale bar: 10 µm.

The ability of MEL and its derivatives to induce DNA double-strand breaks (DSBs) was tested by an immunostaining assay (Figure 3). Cells irradiated with 1 Gy were used as a positive control in the staining process. All tested cell lines were sensitive to 1 Gy irradiation. All compounds, including the original drug MEL, led to a significant increase in γH2AX foci (cut off: 5 foci per cell, *p* < 0.001) in all three cell lines (THP1, HL60, RPMI8226) in relation to each untreated control (Table 1). In the case of THP1, the greatest changes were noticed after 24 h incubation with EM-T-MEL and 48 h incubation with EE-MEL, EM-MEL, EM-I-MEL, and EM-T-MEL. When compared to the original drug MEL, the greatest changes were noticed after 24 h incubation with EM-T-MEL. The 48 h incubation with all compounds led to a statistically significant (*p* < 0.001) increase in γH2AX foci compared to the control. This effect was strongly visible for EE-MEL, EM-MEL, EM-I-MEL, and EM-T-MEL (all drugs except EM-MOR-MEL). The HL60 cells were overall more sensitive than the THP1 cells. The damage was already apparent after 4 h for all drugs in relation to the untreated cells. HL60 showed a higher sensitivity to the modified drugs than the original MEL compound. Significant changes were observed after 4 h treatment with EM-T-MEL, 24 h treatment with all analogs, and 48 h treatment with all tested analogs except EM-I-MEL. RPMI8226 cells responded similarly to THP1 when compared to the untreated controls. When compared to the original MEL, the increase in the number of foci reached a maximum after 4 h and 24 h with EE-MEL, and 24 h with EM-T-MEL. The most explicit effect of an approximately 5.5-fold increase in the abundance of foci was observed after 24 h incubation with EM-T-MEL.

### 2.4. Melphalan and Its Derivatives Induce G2/M Arrest

**Figure 4:**
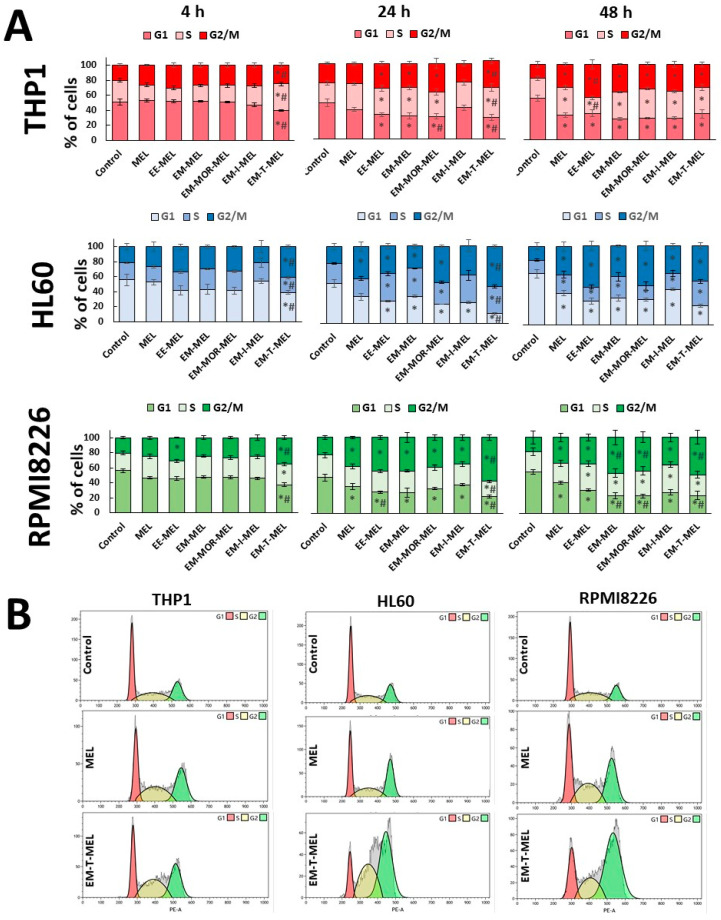
Melphalan and its derivatives induced cell cycle arrest. HL60, THP1, and RPMI8226 cells were incubated for 4, 24, and 48 h with MEL, EE-MEL, EM-MEL, EM-MOR-MEL, EM-I-MEL, and EM-T-MEL. (**A**) Distribution of the cell cycle phases. All data are from three biological assays and are graphed as the mean ± SD. (*) Statistically significant differences compared to the control cells, *p* < 0.05. (#) Statistically significant differences compared to unmodified MEL, *p* < 0.05. (**B**) Representative histograms of cell cycle analysis.

The cell cycle was measured by propidium iodide DNA staining using a flow cytometric technique. The results are shown in Figure 4. Changes in the distribution for each stage were time and compound dependent. We revealed that the greatest effect was induced by EM-T-MEL.

Treatment with EM-T-MEL significantly (*p* < 0.05) changed the distribution of the cell cycle compared to the untreated cells. Compared with the unmodified drug, the time-dependent incubation of cells with EM-T-MEL had the greatest effect on the RPMI8226 cell line, followed by the THP1 and HL60 cells. There was an accumulation of cells in the G2/M phase. In the THP1 cells, there was also an accumulation of cells in the S phase. It is worth noting that changes for this derivative were observed after 4 h of incubation. The level of cells arrested in G2/M after 24 h and 48 h increased by about 2-fold compared to the controls. HL-60 cells in this phase constituted 52% of the cell population at 24 h and 45% at 48 h. This was similar to RPMI8226 cells, in which 58% of the cell population at 24 h and 49% at 48 h, were in the G2/M phase. Simultaneously, the G1 cell population decreased, thereby causing a reversal of the cell cycle profile. In the case of THP1, there was a significant (*p* < 0.05) increase in the G2/M fraction (24 h: 35% of cells; 48 h: 31% of cells) as well as the S fraction (24 h: 40% of cells, 48 h: 35% of cells) at the expense of the G1 phase. Minor but significant (*p* < 0.05) changes in the distribution of the cell cycle were also observed after treating the cells with EE-MEL, EM-MEL, MOR-MEL (24 h, 48 h), and EM-I-MEL (after 48 h). Melphalan, compared to EM-T-MEL, caused less enrichment of cells in the G2/M phase (<30% in HL60 and THP; about 36% in RPMI8226).

### 2.5. Tested Compounds Activate Caspase 2 in a Multiple Myeloma Cell Line

**Figure 5:**
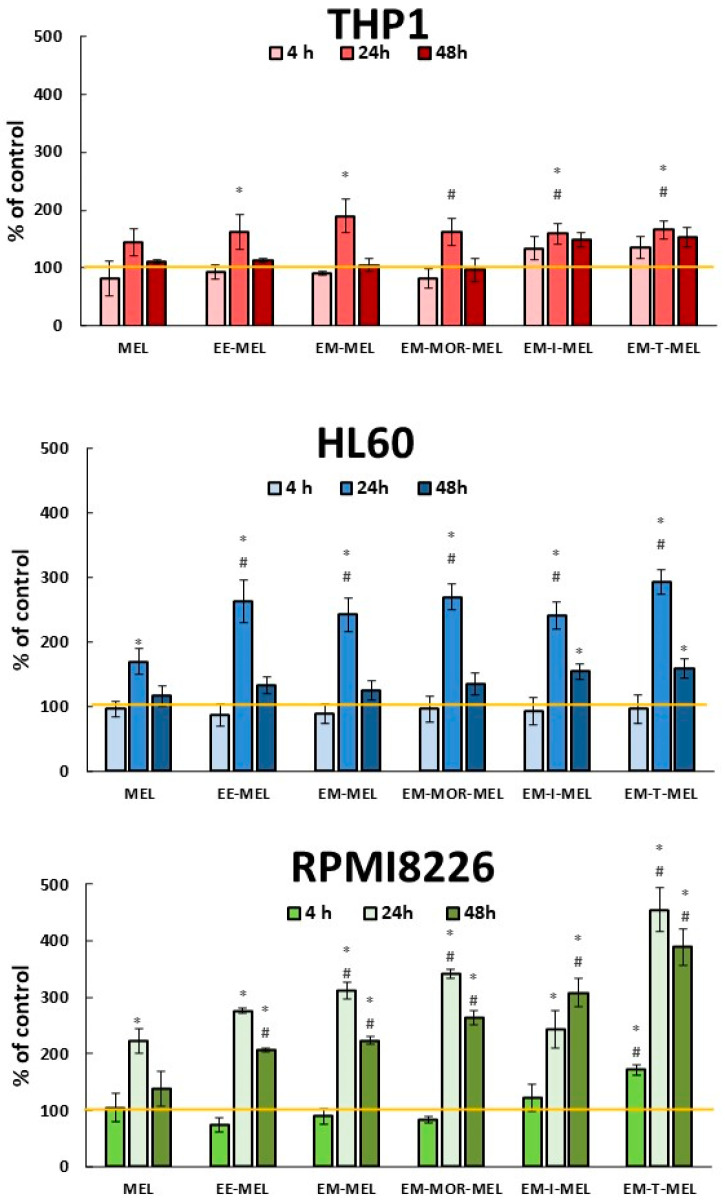
Melphalan derivatives-induced caspase 2 activation. HL60, THP1, RPMI8226 cells were incubated for 4, 24, and 48 h with MEL, EE-MEL, EM-MEL, EM-MOR-MEL, EM-I-MEL, and EM-T-MEL. The final results are expressed as the percentage of activity of a specific cysteine protease, with the untreated control taken as 100%. All data are from three biological assays, and are graphed as the mean ± SD. (*) Statistically significant differences compared to the control cells, *p* < 0.05. (#) Statistically significant differences compared to MEL at the same time point, *p* < 0.05.

Caspase 2 plays an important role in initiating the mitotic catastrophe process. The final results are expressed as a percentage of their activity, with the fluorescence value of the untreated control as 100% (Figure 5).

RPMI8226 cells were the most sensitive to increased caspase 2 activity after longer incubation with all tested derivatives when compared to both leukemia cell lines. Only EM-T-MEL caused changes after 4 h. The greatest activation of caspase 2 occurred after 24 h treatment and for all tested drugs. After this time, statistically significant (*p* < 0.005) changes compared to unmodified MEL (222% ± 21) were observed for EM-MEL (311% ± 15), EM-MOR-MEL (342% ± 8), and EM-T-MEL (455% ± 37). Increasing the incubation time with all tested derivatives up to 48 h was not associated with significantly higher caspase 2 activity. Leukemia cells showed less (HL60) or no increased activity (THP) of caspase 2 compared to RPMI8226 cells. In the case of HL60, most changes were observed after 24 h of incubation with all tested drugs. All modified drugs had more effects than the original drug MEL on caspase 2.

## 3. Discussion

The discovery of new drugs and their pharmacological evaluation is a constant research task of modern science and technology. Understanding the mechanisms of drug actions and their clinical implications is gaining increasing attention in the field of potent drug discovery. Elucidating the mechanisms by which cytotoxic drugs inhibit cancer cell proliferation and induce cell death is essential for optimizing therapeutic efficacy. It is also the basis for the design of new drugs.

In our previous research [14,15], we focused on the synthesis and preliminary biological analysis of new melphalan derivatives with enhanced anti-cancer properties. These studies allowed us to select the five most promising derivatives: melphalan ethyl ester (EE-MEL), melphalan methyl ester (EM-MEL), and melphalan methyl esters with an additional modification in the amine group, which was replaced with an amidine group containing a ring of morpholine (EM-MOR-MEL), indoline (EM-I-MEL), or thiomorpholine (EM-T-MEL). All these derivatives showed higher cytotoxic, genotoxic, and pro-apoptotic properties against multiple myeloma cells (RPMI8226) and leukemic cells (THP1, HL60) than melphalan. In this study, we elucidated the modes of action of selected analogs on tumor cell DNA compared to the original drug melphalan.

Physicochemical methods are key approaches to understanding drug—macromolecule interactions. DNA and proteins are important biomolecules responsible for all necessary cellular metabolism in the biological system. Importantly, interaction studies provide insight into the structure activity relationship of a drug, which is important in designing more effective drugs [21]. CD spectral changes for DNA in the presence of increasing amounts of MEL and its analogs were recorded to gain detailed insight into the mechanism of interaction with DNA. The helix with the right-handed β-C/N-glycosidic bonds and asymmetric DNA pattern in the B form gave a characteristic CD spectrum with a positive band at 280 nm (attributable to the base alignment) and a negative band at 245 nm (due to the right-handed DNA helix) [22,23]. Variations in typical band positions and ellipticities are indicative of suitable conformational transitions in the DNA double helix, due to its interaction with the drug [18]. DNA has three different binding sites: groove binding, binding to a phosphate group, and intercalation [22]. A classical intercalator aligns axially between the DNA base pairs, which in turn leads to an increase in the length of the DNA strand and, thus, to a change in the spiral. This increases the intensity of the positive and negative DNA bands with a shift to higher wavelengths [22,23,24]. On the other hand, the groove binders do not show any shifts or changes in the intensity of the positive and negative bands [25].

In the presence of all tested compounds, a decrease in the intensity of the positive peak with a simultaneous shift to higher waves and an increase in the intensity of the negative peak were observed. The largest changes were observed for EM-T-MEL. The significant reduction in ellipticity in the negative band (245 nm) may have been associated with the destabilization and unwinding of the spiral [26]. For EM-MOR-MEL, a decrease in the intensity of the positive and negative peaks was observed. The shift to higher wavelengths is attributed to conformational transitions from the B to A form, and is additionally associated with decreased ellipticity. This indicates that the complexes modify the base alignment of DNA without inducing significant changes in supramolecular helicity [25]. Due to large changes in ellipticity for EM-MOR-MEL and EM-T-MEL, we can assume that there was partial intercalation [23,27]. Our research showed that unmodified MEL interacted with DNA in a non-intercalation binding mode. It is known that the major site of the alkylation of melphalan is guanine N7 in the major groove [28]. Studies by Bielawska et al. have shown that amidine analogs of melphalan bound to AT-rich sequences in the minor grooves [29].

The results of the circular dichroism analyses were confirmed using other biophysical methods, such as zeta potential measurements and the dynamic light scattering technique. It was shown that melphalan and the tested analogs did not cause the formation of conjugates with DNA because both MEL and all tested analogs were negatively charged.

The cellular response to DNA damage involves a complex network of signaling pathways that lead to cell cycle arrest or cell death [30]. One of these events is the phosphorylation of the histone H2AX to form γH2AX [31]. In eukaryotes, DNA is packed into nucleosomes whose core is an octameric particle consisting of two histones of the classes H2A, H2B, H3, and H4. H2AX is a secondary component of the histone H2A. H2AX phosphorylation is a marker of a DNA double-strand break. Within minutes of DSB formation, several thousand H2AX near the DSB site are phosphorylated at serine 139 to form foci in the nucleus that are microscopically visible by immunofluorescence staining [32]. The results of other researchers have shown that γH2AX foci formation can be used as a pharmacodynamic marker of DNA inter-strand cross-link formation for both nitrogen mustard and platinum-based drugs [33,34]. Our in vitro studies showed that the response to DNA damage in the form of γH2AX foci formation after treatment with the tested compounds was time-dependent for all three cell lines (RPMI8226, H60, and THP1) investigated.

The statistically significant highest ability to phosphorylate histone H2AX was demonstrated by EM-T-MEL, which showed considerably better effects than the original MEL after 24 h and 48 h for THP1; after 4 h, 24 h, and 48 h for HL60; and after 24 h for RPMI8226 cells. These results were consistent with our previous assessment of the levels of DNA damage from these derivatives in the comet test [14,15]. Studies by other groups [35] have confirmed that melphalan is responsible for an increase in γH2AX levels and the induction of phosphorylation of checkpoint kinase 1 (CHK-1) and checkpoint kinase 2 (CHK-2) in MM cells (RPMI8226 and MM1.S).

The antiproliferative properties of antitumor compounds result from their ability to inhibit the division cycle of neoplastic cells. As a result of the genotoxic effects of MEL and the new analogs, the DNA damage response pathway was activated. There was an accumulation of cells in the S- and G2/M phases at the expense of the G1 phase. Studies by other scientists [35,36] have indicated that after treatment with alkylating drugs, including MEL, the most affected phase in terms of cell progression (cytostatic effect) was G2/M [36,37]. Changes in the cell cycle distribution for the tested cell lines depended on the time and sensitivity to the test compound. Treatment with EM-T-MEL significantly affected the distribution of the cell cycle, leading to an accumulation of RPMI8226 and HL60 in the G2/M phase and THP1 additionally in the S-phase at four hours after treatment. Dysfunction of the S-phase checkpoint is one of the main causes of mitotic catastrophe [38]. For the other analogs, the shifted distribution of the cell cycle occurred later: between 24–48 h for EE-MEL, EM-MEL, and MOR-MEL, and after 48 h for EM-I-MEL. Overall, compared to EM-T-MEL, the original drug caused much less enrichment of cells in the G2/M phase.

Our previous studies [14,15] have shown that MEL derivatives exhibited strong pro-apoptotic properties, mainly in leukemic cells (THP-1 and HL-60). The assessment was based on numerous analyses of cell morphological and biochemical changes. THP1 and HL60 cells were particularly sensitive to caspase 3 activation via the mitochondrial apoptosis activation pathway, as opposed to multiple myeloma cells (RPMI8226). It was also shown previously that cells of the RPMI8226 line showed the characteristics of late stages of apoptosis. However, they were least sensitive to caspase 3 activation. No increases in the activation levels of caspase 8 or 9 were observed. This may have indicated the initiation of other molecular mechanisms leading to cell death. All of the tested derivatives induced an accumulation of RPMI8226 cells in the G2/M phase. Cell cycle arrest in this phase is related to the mitotic catastrophe process [38,39] due to defects in the mitotic apparatus, DNA damage, and mitotic checkpoint errors that make complete mitosis impossible. This process is not a typical form of programmed cell death. Other scientists have contributed to the development of mitotic catastrophe by looking at various types of cell death (apoptosis, autophagy, and necrosis), as well as the effect of therapeutic agents acting directly on the DNA [38,40]. The initiation of mitotic catastrophe leads to morphological and biochemical changes in the cells [41]. Cells unable to complete mitosis are characterized by an abnormal increase in cyclin B1 levels [42,43], and are delayed in the G2/M transition, leading to nuclear changes. Multiple nuclei, macronuclei, and micronuclei are formed as a result of chromosome scattering, chromosome breaks, and the disruption of karyokinesis in the metaphase. Reconfiguration of the mitochondrial network can also be considered a morphological feature of mitotic catastrophe [38]. In addition to morphological changes in cell nuclei and mitochondria, the state of mitotic catastrophe is also characterized by an accumulation of H2AX, a biochemical marker of DNA damage [38,44]. Cells undergoing mitotic catastrophe do not show DNA fragmentation that is typical of apoptosis or DNA breaks detected by terminal deoxynucleotidyl transferase biotin-dUTP nick end labeling (TUNEL). Our previous studies [14] for DNA damage analysis (using the TUNEL method) showed a poor response after incubation with all tested compounds, as well as a lack of DNA fragmentation into segments equal to multiples of nucleosome length [42] typical for “classic” apoptosis [45]. This encouraged us to continue the investigations on the mitotic catastrophe process.

One of the most important regulators of mitotic catastrophe is caspase 2 [38,46,47]. The outcome/ending of mitotic catastrophe may be related to the molecular profile of the cell [48]. Thus, to activate this caspase, PIDDosome is formed, which consists of three proteins: the p53-induced death domain protein (PIDD), the RIP-associated Ich-1/Ced-3 homologous protein with a death domain (RAIDD), and caspase 2 [49]. On the other hand, caspase 2 participates in cell cycle regulation by stabilizing p53 and cleaving its inhibitor Mdm2, which are essential for the cellular response during abnormal chromosome segregation and the development of mitotic catastrophe [38]. Melphalan has been shown to activate mitotic catastrophe in RPMI8226 cells [37]. Our study on caspase 2 activation in multiple myeloma cells and leukemic cells showed a significant increase in caspase activity, mainly after 24 h incubation. The RPMI8226 cell line turned out to be the most sensitive. The greatest changes were observed after incubation with EM-T-MEL.

Mitotic catastrophe is characterized by nuclear changes that lead to multinucleation and/or micronucleation [38,42]. Under conditions of mitotic catastrophe, changes occur in the cell nucleus, in which the mitochondria are also involved. Changes in the mitochondrial network have been shown to accelerate the development of mitotic catastrophe [38,41,48]. The induction of mitotic catastrophe by doxorubicin leads to the remodeling of mitochondrial structure and the fragmentation of mitochondria in colon cancer cells. Moreover, in breast cancer cells, mitochondrial fission promotes radiation-induced mitotic catastrophe and increases Ca^2+^ levels in the cytosol [38].

In the present study, we focused on another important regulator of mitotic catastrophe, caspase 2, which is involved in maintaining genome stability. Polyploid and aneuploid cells are prone to mitotic catastrophe. As part of the PIDDosome complex, caspase 2 participates in the elimination of extra centrosomes and regulates ploidy and cell proliferation. In addition, caspase 2 activates the PCD process in cells with abnormal numbers of chromosomes and centrosomes, which also prevents the development of mitotic catastrophe [38]. Caspase 2 deficiency results in the predisposition of cancer cells to aneuploidy, due to B-cell CLL/lymphoma 9-like (BCL9L) protein dysfunction [38,50]. Our results showed high caspase 2 activity after treatment with new highly genotoxic analogs, mainly in multiple myeloma cells, which indicated high genomic instability. Based on the data, we concluded that high caspase 2 activity may be associated with the removal of cells with mitotic aberrations to reduce aneuploidy, so we did not expect a large number of cells with a mitotic catastrophe phenotype. The reconfiguration of the mitochondrial network can also be considered a morphological feature of mitotic catastrophe [38]. Our previous data [14,15] showed that mitochondrial membrane potential decreased after treatment with melphalan analogs in cancer cell lines. We also observed subtle increases in the activation of the exocrine caspase 3. Based on the current and previous studies, we can suspect that one of the pathways of multiple myeloma cell death may be mitotic catastrophe [41,42,48].

## 4. Materials and Methods

### 4.1. Materials

#### 4.1.1. Melphalan Derivatives

**Figure 6:**
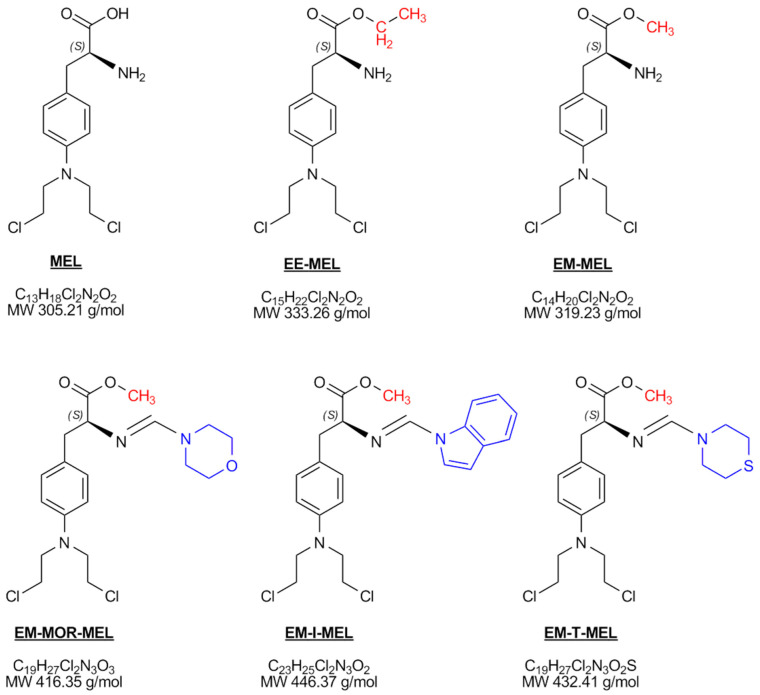
Chemical structure of melphalan (MEL) and its tested derivatives.

This study used MEL (2S)-2-amino-3-[4-[bis(2-chloroethyl)amino]phenyl]propanoic acid and its five analogs: EE-MEL (2S)-2-amino-3-[-4-[bis(2-chloroethyl)amino]phenyl] propanoic acid ethyl ester; EM-MEL (2S)-2-amino-3-[-4-[bis (2-chloroethyl)amino]phenyl] propanoic acid ethyl ester; EM-MOR-MEL (2S)-2-(morpholinmethylideneamino)-3-[4-[bis(2-chloroethyl)amino]phenyl] propanoic acid methyl ester; EM-I-MEL (2S)-2-(indolinmethylideneamino)-3-[4-[bis(2-chloroethyl)amino]phenyl] propanoic acid methyl ester; and EM-T-MEL (2S)-2-(thiomorpholinmethylideneamino)-3-[4-[bis(2-chloroethyl)amino]phenyl] propanoic acid methyl ester (Figure 6). Their synthesis has been reviewed in our previous publications [14,15].

#### 4.1.2. Cell Culture, Drug Concentration, and Treatment Time

The acute monocytic leukemia cell line THP1 (ATCC^®^ TIB–202™), promyelocytic leukemia cell line HL60 (ATCC^®^ CCL–240™), and the multiple myeloma cell line RPMI8226 (ATCC^®^ CCL–155™) were obtained from the American Type Culture Collection (ATCC, Rockville, MD, USA). All the investigated cells were cultured in RPMI 1640 medium supplemented with 10% fetal bovine serum, penicillin (10 U/mL), and streptomycin (50 μg/mL) in standard conditions: 37 °C, 100% humidity, and an atmosphere of 5% CO_2_ and 95% air. Cell viability was systematically controlled using 0.4% trypan blue. In all experiments, cells in a logarithmic growth phase were used when their viability was above 95%.

One concentration of the drugs for each cell line was chosen for the study: THP1: 0.3 µM; HL60: 0.7 µM; RPMI8226: 3 µM. These were the same as in previous studies evaluating the biological properties of melphalan analogs [14,15]. In all experiments, cells were treated for 4 h, 24 h, and 48 h. Simultaneously, control cultures were incubated similarly without further treatment. For the immunostaining assay, a positive control from irradiated cells was prepared. The cells were irradiated with 1 Gy of ionizing radiation by an ISOVOLT Titan X-ray generator (GE, Ahrensburg, Germany) [51,52].

### 4.2. Methods

#### 4.2.1. Circular Dichroism as a Technique for the Analysis of the Alteration of DNA Conformation

The circular dichroism spectra of deoxyribonucleic acid from the calf thymus were measured using a J-815CD spectrometer (Jasco, Japan). Complexes of DNA/MEL or MEL derivatives were prepared in a 10 mM Na-phosphate buffer, with a pH of 7.4. The concentration of DNA in the samples was 250 µg/mL. However, the concentrations of compounds in the samples were 5 µM, 25 µM, 50 µM, 75 µM, 100 µM, 150 µM, 200 µM, 250 µM, and 300 µM. The measurements were made in a Helma quartz cell with a thickness of 0.5 cm. The scan parameters were as follows: wavelengths of 230–320 nm; scan speed of 100 nm/min, and bandwidth of 1.0 nm. The slit was set on the auto mode, and n = 3. The mean ellipticity was calculated using software provided by Jasco.

#### 4.2.2. Zeta Potential Measurement and DLS for Measuring the Hydrodynamic Diameters of the Particles

Zeta potential was measured using laser Doppler velocimetry by Zetasizer Nano ZS-90 (Malvern Instruments, United Kingdom) and calculated using the Smoluchowski equation. Complexes of DNA/MEL or MEL derivatives were prepared in a 10 mM Na-phosphate buffer, with a pH of 7.4. The concentration of DNA in the samples was 30 µg/mL. The concentrations of compounds in the samples were 2.5 µM, 5 µM, 10 µM, 25 µM, 50 µM, and 100 µM. Measurements were made with or without DNA. Data analysis was performed using Malvern software and given as the mean ± standard deviation (SD) obtained from five measurements in seven cycles at room temperature (RT) for each sample.

Hydrodynamic diameters of the complexes were measured by the DLS method in a photon correlation spectrometer (Zetasizer Nano ZS-90, Malvern Instruments, Malvern, UK). The wavelength was set at 633 nm, the refraction factor was 1.33, and the detection angle was 900. Complexes of DNA/MEL or MEL derivatives were prepared in a 10 mM Na-phosphate buffer, with a pH of 7.4. Measurements were made with or without DNA. The data analysis was performed using Malvern software and is given as the mean ± SD obtained from five measurements in five cycles at RT for each sample.

#### 4.2.3. Immunofluorescence Staining of γH2AX for DNA Damage Detection

The essence of the test was the detection and localization of γH2AX histone foci, based on the highly specific antigen–antibody binding reaction. Based on previous publications, a protocol for fixing [53,54] and staining [55,56] non-adherent cells was prepared.

Cells were treated in Petri dishes with all tested compounds, as described above. Additionally, a positive control was prepared in which the cells irradiated with 1 Gy. Cells were washed with PBS (300 rpm, 5 min, RT), spotted on the surface of defatted glass slides, and incubated for 10 min to adhere cells to the coverslips. Adherent cells were fixed in 4% formaldehyde solution with 0.1% Triton X-100. Permeabilized cells were washed with Tris-buffered saline (TBS) and blocked with 1% BSA in PBS overnight. After incubation and washing with TBS, staining was performed. Cells were incubated with a phosphorylated (Ser139) mouse monoclonal IgG-specific anti-H2AX antibody at a dilution of 1:2500 (BioLegend, San Diego, United States) suspended in TBS buffer supplemented with 1% BSA. Incubation was for 1 h in the dark in a humid chamber at 24 °C. Then, a washing step with TBS was performed. The cells were then incubated with Alexa Fluor TM 488 goat anti-mouse IgG secondary antibody at a dilution of 1:400 (Thermo Fisher Scientific, Waltham, MA, USA) suspended in TBS buffer supplemented with 1% BSA. Incubation was for 3 h in the dark in a humid chamber at 24 °C. The cell nuclei were then stained with 4′,6-diamidino-2-phenylindole (DAPI) solution (0.1 ng/mL). Microscopic analysis (Zeiss Axio Plan 2, Göttingen, Germany) was started, and digital recordings were taken using the MetaSystem software (Altlussheim, Germany). The photos were analyzed with ImageJ (LOCI, University of Wisconsin). The number of foci in each cell was counted; 100 cells were counted from each image.

#### 4.2.4. Flow Cytometry for Cell Cycle Analysis

Flow cytometry was used to quantify the cellular DNA content. Cells were treated as described above. After incubation, cells were harvested, washed with PBS, and fixed in 70% ethanol (minimum 24 h; 4 °C). Following ethanol fixation, the cells were washed in PBS and centrifuged at 7000× *g* for 10 min at 4 °C. Harvested cells were stained by adding 300 µL of PBS containing PI and RNase at final concentrations of 75 µM and 20 µg/mL, respectively. The samples were incubated for 1 h in complete darkness at 37 °C. The stained cells were analyzed using flow cytometry (Becton Dickinson, San Jose, CA, USA), and the percentage of cells in each cell phase was calculated [57].

#### 4.2.5. Activity of Caspase 2

The test was based on detecting the cleavage of substrate VDVAD (Val-Asp-Val-Ala-Asp)-AFC (7-amino-4-trifluoromethycoumarin). VDVAD-AFC emits blue light (λ = 400 nm). However, after substrate cleavage by caspase 2, free AFC emits yellow-green fluorescence (λ = 505 nm), which can be quantified with a fluorometer plate reader. The activities of caspase 2 were estimated with a caspase 2 assay kit (Fluorometric) according to the manufacturer’s protocols (Abcam, Cambridge, UK).

Cells were treated on Petri dishes with tested compounds as described above. Next, cells were counted and placed in 96-well black fluorometric plates (1 × 10^6^/well). Cells were resuspended in 50 µL of chilled cell lysis buffer and incubated on ice for 10 min. Reaction buffer (50 µL) containing 10 mmol of DTT and 5 µL of the 1 mM VDVAD-AFC substrate was added to each well and incubated for 2 h at 37 °C. The measurement was done on a Fluoroscan Ascent FL plate reader (Labsystem, Stockholm, Sweden) using a 400 nm excitation filter and a 505 nm emission filter. Caspase 2 activity was expressed as a ratio of fluorescence of the treated sample relative to the corresponding untreated control, which was taken as 100%.

#### 4.2.6. Statistical Analysis

Data are presented as the mean ± standard deviation (SD) unless indicated otherwise.

For analyzing changes from the baseline (control cell culture without treatment or the original drug MEL), the analysis of variance (ANOVA) with Tukey’s post hoc test was used. The data from two independent biological assays (100 cells counted in each case, n = 200) were merged for the immunostaining analysis. Foci-positive cells were labeled as cells with more than five foci according to or based on a previous study [32]. The statistical analyses were performed using STATISTICA (StatSoft, Tulsa, OK, USA). The level of significance for all analyses was set at α = 0.05 (two-tailed). All figures include descriptions of statistically significant changes: * *p* < 0.05 signifies a statistically significant difference compared to control cells, and # *p* < 0.05 signifies a statistically significant difference compared to MEL.

## 5. Conclusions

In this study, we proved that tumor cell DNA is the main molecular target for the chemically modified analogs of the widely used anti-cancer drug melphalan.

The newly synthesized derivatives, in particular EM-MOR-MEL and EM-T-MEL, affected the B-DNA conformation, which increased the DNA damage detected by γH2AX analysis. As a result of the DNA changes, the cell cycle was arrested in the S and G2/M phases. The cell death occurred by activating a mitotic catastrophe as a special example of apoptosis. Our investigations suggest that the analogs EM-MOR-MEL and EM-T-MEL have better anti-cancer activity in multiple myeloma cells than the currently used melphalan, at least in the in vitro models applied.

## Figures and Tables

**Table 1:** γH2AX foci per cell after treatment with melphalan and its derivatives. Data are the number of foci per cell (%). All data are from two biological assays (n = 200; 100 cells counted in each case). Statistically significant *p* values (ANOVA with Tukey’s *post hoc*) for cells > 5 foci/cell compared to the control or original MEL drug are shown.

THP1 (n = 200)								
4 h								
foci/cell	Control	1 Gy	MEL	EE_MEL	EM-MEL	EM_MOR_MEL	EM_I_MEL	EM_T_MEL
0–5	155 (77.5%)	30 (15%)	73 (36.5%)	63 (31.5%)	70 (35%)	109 (54.5%)	87 (43.5%)	59 (29.6)
6–10	22 (11%)	50 (25%)	69 (34.5%)	40 (20%)	43 (21.5%)	29 (14.5%)	43 (21.5%)	47 (23.6%)
11–19	5 (2.5%)	42 (21%)	12 (6%)	43 (21.5%)	25 (12.5%)	28 (14%)	23 (11.5%)	32 (16.1%)
≥20	11 (5.5%)	45 (22.5%)	31 (15.5%)	31 (15.5%)	45 (22.5%)	27 (13.5%)	36 (18%)	36 (18.1%)
an abundance of foci	7 (3.5%)	33 (16.5%)	15 (7.5%)	23 (11.5%)	17 (8.5%)	7 (3.5%)	11 (5.5%)	25 (12.6%)
compared to control		*p* = 0.000177	*p* = 0.000177	*p* = 0.000177	*p* = 0.000177	*p* = 0.000177	*p* = 0.000177	*p* = 0.000177
compared to MEL		*p* = 0.000177						
24 h								
0–5	152 (76%)		43 (21.5%)	34 (17.1%)	30 (15%)	28 (14%)	64 (32%)	16 (8%)
6–10	45 (22.5%)		56 (28%)	48 (24.1%)	34 (17%)	33 (16.5%)	47 (23.5%)	25 (12.5%)
11–19	3 (1.5%)		40 (20%)	43 (21.6%)	39 (19.5%)	36 (18%)	36 (18%)	29 (14.5%)
≥20	0 (0%)		49 (24.5%)	44 (22.1%)	59 (29.5%)	57 (28.5%)	49 (24.5%)	66 (33%)
an abundance of foci	0 (0%)		12 (6%)	30 (15.1%)	38 (19%)	46 (23%)	4 (2%)	64 (32%)
compared to control			*p* = 0.000177	*p* = 0.000177	*p* = 0.000177	*p* = 0.000177	*p* = 0.000177	*p* = 0.000177
compared to MEL								*p* = 0.000374
48 h								
0–5	118 (59%)		43 (21.5%)	11 (5.5%)	24 (12%)	30 (15%)	15 (7.5%)	9 (4.5%)
6–10	50 (25%)		50 (25%)	16 (8%)	39 (19.5%)	22 (11%)	39 (19.5%)	23 (11.5%)
11–19	19 (9.5%)		40 (20%)	17 (8.5%)	60 (30%)	35 (17.5%)	40 (20%)	31 (15.5%)
≥ 20	8 (4%)		42 (21%)	67 (33.5%)	54 (27%)	64 (32%)	57 (28.5%)	59 (29.5%)
an abundance of foci	5 (2.5%)		25 (12.5%)	89 (44.5%)	23 (11.5%)	49 (24.5%)	49 (24.5%)	78 (39%)
compared to control			*p* = 0.000177	*p* = 0.000177	*p* = 0.000177	*p* = 0.000177	*p* = 0.000177	*p* = 0.000177
compared to MEL				*p* = 0.000180	*p* = 0.034157		*p* = 0.000271	*p* = 0.000177
HL60 (n = 200)								
4 h								
foci/cell	Control	1 Gy	MEL	EE_MEL	EM-MEL	EM_MOR_MEL	EM_I_MEL	EM_T_MEL
0–5	180 (90.5%)	19 (9.5%)	130 (65%)	140 (70%)	126 (63%)	117 (58.5%)	127 (63.5%)	90 (45%)
6–10	14 (7%)	62 (31%)	45 (22.5%)	39 (19.5%)	42 (21%)	50 (25%)	29 (14.5%)	37 (18.5%)
11–19	2 (1%)	84 (42%)	11 (5.5%)	10 (5%)	16 (8%)	15 (7.5%)	28 (14%)	24 (12%)
≥ 20	3 (1.5%)	18 (9%)	13 (6.5%)	11 (5.5%)	8 (4%)	17 (8.5%)	15 (7.5%)	37 (18.5%)
an abundance of foci	0 (0%)	17 (8.5%)	1 (0.5%)	0 (0%)	8 (4%)	1 (0.5%)	1 (0.5%)	12 (6%)
compared to control		*p* = 0.000177	*p* = 0.000177	*p* = 0.000177	*p* = 0.000177	*p* = 0.000177	*p* = 0.000177	*p* = 0.000177
compared to MEL		*p* = 0.000177						*p* = 0.000177
24 h								
0–5	150 (75%)		104 (52.5%)	23 (11.6%)	37 (18.7%)	60 (30%)	71 (35.5%)	37 (18.8%)
6–10	39 (19.5%)		41 (20.7%)	32 (16.2%)	32 (16.2%)	39 (19.5%)	44 (22%)	33 (16.8%)
11–19	9 (4.5%)		23 (11.6%)	20 (10.1%)	24 (12.1%)	31 (15.5%)	21 (10.5%)	29 (14.7%)
≥ 20	2 (1%)		21 (10.6%)	88 (44.4%)	68 (34.3%)	50 (25%)	53 (26.5%)	58 (29.4%)
an abundance of foci	0 (0%)		9 (4.5%)	35 (17.7%)	37 (18.7%)	20 (10%)	11 (5.5%)	40 (20.3%)
compared to control			*p* = 0.000177	*p* = 0.000177	*p* = 0.000177	*p* = 0.000177	*p* = 0.000177	*p* = 0.000177
compared to MEL				*p* = 0.000177	*p* = 0.000177	*p* = 0.000177	*p* = 0.000177	*p* = 0.000177
48 h								
0–5	182 (91%)		53 (26.5%)	11 (5.6%)	18 (9%)	8 (4%)	57 (28.5%)	37 (18.5%)
6–10	18 (9%)		22 (11%)	17 (8.6%)	26 (13%)	19 (9.5%)	18 (9%)	27 (13.5%)
11–19	0 (0%)		13 (6.5%)	23 (11.7%)	27 (13.5%)	33 (16.5%)	16 (8%)	19 (9.5%)
≥ 20	0 (0%)		54 (27%)	61 (31%)	61 (30.5%)	58 (29%)	61 (30.5%)	52 (26%)
an abundance of foci	0 (0%)		58 (29%)	85 (43.1%)	68 (34%)	82 (41%)	48 (24%)	65 (32%)
compared to control			*p* = 0.000177	*p* = 0.000177	*p* = 0.000177	*p* = 0.000177	*p* = 0.000177	*p* = 0.000177
compared to MEL				*p* = 0.000177	*p* = 0.000177	*p* = 0.000177		*p* = 0.005658
RMPI8226 (n = 200)								
4 h								
foci/cell	Control	1 Gy	MEL	EE_MEL	EM-MEL	EM_MOR_MEL	EM_I_MEL	EM_T_MEL
0–5	155 (77.5%)	30 (15%)	73 (36.5%)	63 (31.5%)	70 (35%)	109 (54.5%)	87 (43.5%)	59 (29.6%)
6–10	22 (11.0%)	50 (25%)	69 (34.5%)	40 (20%)	43 (21.5%)	29 (14.5%)	43 (21.5%)	47 (23.6%)
11–19	5 (2.5%)	42 (21%)	12 (6%)	43 (21.5%)	25 (12.5%)	28 (14%)	23 (11.5%)	32 (16.1%)
≥ 20	11 (5.5%)	45 (22.5%)	31 (15.5%)	31 (15.5%)	45 (22.5%)	27 (13.5%)	36 (18%)	36 (18.1%)
an abundance of foci	7 (3.5%)	33 (16.5%)	15 (7.5%)	23 (11.5%)	17 (8.5%)	7 (3.5%)	11 (5.5%)	25 (12.6%)
compared to control		*p* = 0.000177	*p* = 0.000177	*p* = 0.000177	*p* = 0.000177	*p* = 0.000177	*p* = 0.000177	*p* = 0.000177
compared to MEL				*p* = 0.011650				
24 h								
0–5	152 (76%)		43 (21.5%)	34 (17.1%)	30 (15%)	28 (14%)	64 (32%)	16 (8%)
6–10	45 (22.5%)		56 (28%)	48 (24.1%)	34 (17%)	33 (16.5%)	47 (23.5%)	25 (12.5%)
11–19	3 (1.5%)		40 (20%)	43 (21.6%)	39 (19.5%)	36 (18%)	36 (18%)	29 (14.5%)
≥ 20	0 (0%)		49 (24.5%)	44 (22.1%)	59 (29.5%)	57 (28.5%)	49 (24.5%)	66 (33%)
an abundance of foci	0 (0%)		12 (6%)	30 (15.1%)	38 (19%)	46 (23%)	4 (2%)	64 (32%)
compared to control			*p* = 0.000177	*p* = 0.000177	*p* = 0.000177	*p* = 0.000177	*p* = 0.000177	*p* = 0.000177
compared to MEL				*p* = 0.000347				*p* = 0.002221
48 h								
0–5	118 (59%)		43 (21.5%)	11 (5.5%)	24 (12%)	30 (15%)	15 (7.5%)	9 (4.5%)
6–10	50 (25%)		50 (25%)	16 (8%)	39 (19.5%)	22 (11%)	39 (19.5%)	23 (11.5%)
11–19	19 (9.5%)		40 (20%)	17 (8.5%)	60 (30%)	35 (17.5%)	40 (20%)	31 (15.5%)
≥ 20	8 (4%)		42 (21%)	67 (33.5%)	54 (27%)	64 (32%)	57 (28.5%)	59 (29.5%)
an abundance of foci	5 (2.5%)		25 (12.5%)	89 (44.5%)	23 (11.5%)	49 (24.5%)	49 (24.5%)	78 (39%)
compared to control			*p* = 0.000177	*p* = 0.000177	*p* = 0.000177	*p* = 0.000177	*p* = 0.000177	*p* = 0.000177
compared to MEL								

## Data Availability

The datasets presented during in the current study are available from the corresponding author upon reasonable request.
